# Childhood trauma, substance use and depressive symptoms in people with HIV during COVID-19

**DOI:** 10.4102/sajpsychiatry.v30i0.2220

**Published:** 2024-10-08

**Authors:** Nikita Prosad Singh, Vuyokazi Ntlantsana, Andrew Tomita, Saeeda Paruk

**Affiliations:** 1 Department of Psychiatry, School of Clinical Medicine, College of Health Sciences, University of KwaZulu-Natal, Durban, South Africa; 2 KwaZulu-Natal Research Innovation and Sequencing Platform (KRISP), College of Health Sciences, University of KwaZulu-Natal, Durban, South Africa; 3 Centre for Rural Health, School of Nursing and Public Health, College of Health Sciences, University of KwaZulu-Natal, Durban, South Africa

**Keywords:** adverse childhood experiences, alcohol, tobacco, cannabis, depression, HIV, COVID-19, South Africa

## Abstract

**Background:**

Adverse childhood experiences (ACEs), substance use, depressive symptoms, and HIV outcomes in people living with HIV (PLWHIV) have not been comprehensively investigated within a single study.

**Aim:**

The aim of this study was to investigate the prevalence and association of ACEs, substance use, depressive symptoms and human immunodeficiency virus (HIV) outcomes in PLWHIV accessing HIV care during the coronavirus disease 2019 (COVID-19) pandemic.

**Setting:**

District hospital HIV clinic in South Africa.

**Methods:**

A total of 196 PLWHIV completed a socio-demographic and clinical questionnaire; an adapted World Health Organization (WHO) Alcohol, Smoking and Substance Involvement Screening Test; WHO ACEs International Questionnaire, and Patient Health Questionnaire.

**Results:**

The most common ACEs were having one or no parent, parental separation or divorce (*n* = 131, 66.8%), exposure to collective (*n* = 57, 29.1%) and community violence (*n* = 55, 28.1%), with 40.3% (*n* = 79) experiencing ≥ 3 ACEs. The most commonly used substances were alcohol (*n* = 34, 17.3%), tobacco (*n* = 33, 16.8%), and cannabis (*n* =13, 6.6%). The prevalence of depressive symptoms was 19.4% (*n* = 38). Linear regression analyses indicated greater alcohol (adj β = 2.84, *p* < 0.01), tobacco (adj β = 3.64, *p* < 0.01) and cannabis use risk scores (adj β = 2.39, *p* < 0.01) were associated with ≥ 3 ACEs. Logistic regression indicated depressive risk (adjusted odds ratio [OR] = 9.39, 95% confidence interval [CI] 4.78–23.51) was associated with ≥ 3 ACEs.

**Conclusion:**

A high prevalence of ACEs, substance use and depressive symptoms exists among PLWHIV, along with an association between cumulative ACEs and both substance use and depressive symptoms.

**Contribution:**

Enhanced screening and management services are recommended to address this triple burden in PLWHIV.

## Introduction

South Africa (SA) has several public health challenges including having a high burden of mental illness,^1^ being the epicentre of HIV epidemic,^2^ and more recently the coronavirus disease 2019 (COVID-19) pandemic with more than 4 million confirmed cases since the onset.^3^ Estimates indicate that at least one in six adult South Africans may be living with a mental health condition in a 12-month period.^1^ Substance use continues to be a problem with approximately 15% of South Africans using drugs.^4^ During 2020 in SA, the estimated HIV prevalence rate in adults aged 15–49 years was 19.1%.^5^

Mental healthcare services in SA are severely limited,^6^ with the COVID-19 pandemic further increasing stress on the overburdened healthcare system, because of increased morbidity and mortality associated with the viral pandemic.^7^ In SA, the COVID-19 pandemic and the subsequent lockdown had significant impacts on mental health with social isolation, limited physical mobility and access to health care, emotional distress and financial insecurity.^6,8^ Pre-existing chronic conditions like cardiovascular diseases are prevalent in PLWHIV, and influence COVID-19 severity. People living with HIV (PLWHIV) are at risk of anaemia, neutropenia, thrombocytopenia and abnormal serum electrolytes, which also play a critical role in the disease course of COVID-19.^9^

The relationship between substance use disorders (SUDs) and mental disorders is known to be bi-directional.^10^ People living with HIV are disproportionately affected by mental health disorders,^11^ with the prevalence of depressive symptoms, substance abuse and a psychiatric diagnosis in PLWHIV being 72.9%.^12^ In previous smaller studies conducted in KwaZulu-Natal (KZN), SA, no association was found between substance use and HIV among individuals living with psychosis, although these studies had limited sample sizes and did not investigate depressive symptoms.^13^

Substance use is a risk factor for acquiring HIV,^14^ and the consumption of substances is higher among PLWHIV than in the general population.^15^ A high prevalence of alcohol use in PLWHIV was found in SA,^16^ with a 12.4% prevalence for alcohol use disorder.^17^ Rates of tobacco use in PLWHIV are nearly twice general population rates,^18^ at 40% – 70% internationally.^19^ Rates of cannabis use are also higher in PLWHIV than the general population with prevalence estimates ranging between 20% and 60%.^20^ Substance abuse has been found to be predictor of poor adherence to highly active antiretroviral therapy (HAART)^21^ and hazardous drinking and active drug use are associated with decreased viral suppression in PLWHIV.^22^

Rates of current depression among PLWHIV are at least twice the general population rates,^21^ a depression prevalence rate of 41% being found in a cohort of PLWHIV in SA.^17^ Prior to the COVID-19 pandemic, the pooled prevalence of depressive symptoms among PLWHIV in sub-Saharan Africa (SSA) was 32%.^11^ Depression is the most important predictor of poor treatment adherence.^23^

Adverse Childhood Experiences (ACEs) constitute a group of potentially traumatic events (abuse, neglect and household dysfunction) that occur during the first 17 years of life.^24^ These experiences are common in young adults from SSA,^25^ with nearly 75% of participants of the South African Stress and Health (SASH) study reporting some traumatic event during their lifetime.^26^ The presence of one ACE significantly increases the likelihood of having additional ACEs,^27^ with individuals in the SASH study found to be experiencing multiple traumas.^26^

There is increasing evidence that PLWHIV experience high levels of ACEs,^15^ often at rates greater than the general population.^28,29^ Adverse childhood experiences are recognised as meaningful predictors of HIV risk,^15^ by increasing sexual behaviour and drug use. Among PLWHIV, increasing ACEs have been associated with poor appointment adherence and higher viral loads (VL).^29^

There is a strong relationship between ACEs and substance use and abuse later in life,^30^ which increases as the ACE score is increased.^31^ Adults who experienced four or more ACEs were at high risk for alcoholism and drug use.^30^ A meta-analysis found that ACEs were associated with more than a two-fold increase in risk for depression in adulthood and may be a significant predictor of depression in adulthood.^32^ A graded dose response relationship was found between an expanded ACE score and the likelihood of a depressed affect in adulthood.^33^ A study exploring the association between ACEs, depression and anxiety symptoms, substance use, and HIV-related outcomes in a sample of 584 PWHIV at risk for unhealthy alcohol use found an association between ACEs and anxiety but no association between ACEs and HIV, depression and substance use outcomes. The mixed results on the association of ACEs with mental health and substance use outcomes suggest that further investigation is warranted.^15^

In an international sample, 13.3% of the respondents reported having started or increased substance use to cope with COVID-19 stress.^7^ However, a study on young adults showed no increases in alcohol use during the COVID-19 pandemic in SA.^25^ According to a review, studies that examined substance use among PLWHIV during early COVID-19 reported variable results with no consistent pattern.^8^ Cigarette use patterns have also varied in response to the COVID-19 pandemic.^34^ Several cross-sectional studies reported mixed results with cannabis use having increased, decreased or remaining unchanged according to a scoping review done during the pandemic.^35^

The pooled prevalence of depression in 2020 was 7-fold the estimated global prevalence in 2017,^36^ and depressive symptoms during the pandemic increased 3-fold compared to pre-pandemic in a sample from North America.^37^ A study on young adults showed increases in depression during the COVID-19 pandemic in SA.^25^ In SA, it has been reported that the relationship between increased depressive symptoms and greater perceived COVID-19 infection risk was more severe among adults who reported worse histories of ACEs.^6^ People living with HIV indicated significantly more depressive symptoms during COVID-19 compared to pre-pandemic.^8^

Significant gaps exist in the management of mental health and SUDs in PLWHIV in developing countries,^38^ and while there is a significant relationship between ACEs, substance use and mental health disorders,^39^ few studies have assessed all these factors in PLWHIV.^22^ Illicit substance use in the last 90 days was reported by 27% of treatment arm participants and 31% of control arm participants in a randomised controlled study of PLWHIV diagnosed with moderate-severe depression at baseline.^40^ Severe depression was reported by 54.7% of a cohort of PLWHIV abusing substances.^41^ The mental health burden in PLWHIV has also been compounded by the public health crisis of COVID-19,^11^ and studies worldwide have highlighted the depressive impacts of psychosocial stressors because of the COVID-19 pandemic.^42^ Unrecognised and untreated depressive and SUDs could have the potential to impact HIV care,^16,32^ and these possible barriers to adherence need to be investigated and managed appropriately.

## Aim

The aim of this study was to investigate the prevalence and association of ACEs, substance use, depressive symptoms and HIV outcomes in PLWHIV accessing HIV care during the COVID-19 pandemic.

## Research methods and design

### Study design and setting

This cross-sectional, quantitative, descriptive study was conducted at a HIV clinic in a public, district-level general hospital in Eastern SA from September to December 2020 after the first wave of the COVID-19 pandemic in SA. The outpatient HIV clinic manages an average of 40 patients a day, 5 days a week and comprises of a diverse multicultural population.

### Sample population, size and sampling strategy

The inclusion criteria used were patients 18 years or older with confirmed HIV infection, on highly active antiretroviral therapy (HAART), attending the HIV clinic and able to read and write in English or isiZulu (local language in KZN). Those who were unwilling or unable to provide consent were excluded. Because of COVID-19 risk, each patient was approached individually by the principal investigator for written informed consent. Data were collected via survey questionnaires that were completed in writing by the participants while awaiting consultation or medication in the clinic. Convenience sampling was utilised by the principal investigator who was solely responsible for data collection. The required sample size calculated was 196, which was determined on the statistical power of 80% and on the basis of the desired significance level of 0.05.

### Data collection tools

The survey consisted of a socio-demographic and clinical questionnaire; an adapted version of the World Health Organization (WHO) Alcohol, Smoking and Substance Involvement Screening Test (ASSIST V 3.0); the WHO Adverse Childhood Experiences International Questionnaire (ACE-IQ), and the Patient Health Questionnaire (PHQ-9), which are all suitable for self-administration by the participants.

The main study predictor was ACEs, measured with the WHO ACE-IQ, a standardised instrument with 13 categories of adversities with good psychometric properties and has also been previously used in SA.^13,43^ The ACE score was computed (ranging from 0 to 13) using the recommended binary version, with an affirmative response being counted as a Yes for that specific ACE category.

Self-report of lifetime substance and current substance use (in the past 3 months) was collected for alcohol, tobacco or tobacco-related products, cannabis, cocaine, amphetamine, inhalants, sedatives and/or sleeping pills, hallucinogens and opioids, as per an adapted version of the WHO ASSIST V 3.0 containing terms applicable to the local context. It has been translated into isiZulu and utilised in SA.^13,44^ Validated cut-off points stratifying scores were as follows: Questions 2–7, a score of 0–3 (0–10 for alcohol) for a specific substance indicated low substance risk involvement and no intervention required; Questions 4–26 (11–26 for alcohol) indicated moderate substance risk involvement and brief intervention required, and scores greater than 27 suggest a high risk of substance dependence with intensive intervention required.

The PHQ-9, a nine-item questionnaire to make criteria-based diagnoses of depressive disorders and grade symptom severity in primary care settings, was used. The questionnaire uses a 4-point Likert scale (0 = not at all, 1 = several days, 2 = more than half the days, 3 = nearly every day) to assess the responses about mental and/or emotional health over the past 2-week period. The standard cut-off score used to identify possible moderate to severe depression of ≥ 10 was used. The PHQ-9 has shown diagnostic validity in SA,^45^ and has been used in SA.^44^

A chart review was conducted to confirm the patients’ HAART regimens and National Health Laboratory Service results were checked to confirm positive HIV diagnosis, clusters of differentiation 4 (CD4) counts and quantitative HIV VL. Virologic detection was defined as a VL measurement of at least 50 copies/mL based on the assay.

### Data analysis

Firstly, descriptive statistics was used to summarise the socio-demographic and clinical profiles of the participants. Secondly, associations between ACE and clinical outcomes (depressive symptoms, substance use and VL) were examined using Pearson χ^2^ statistics. Finally, the above analysis was repeated using linear regression (for substance use scores) and logistic regression (for depression and VL suppression categories), while controlling for socio-demographic characteristics, including age, gender, marital status, residential area, employment, education and CD4 count. Linear regression was chosen for substance use scores, as they were continuous variables, while logistic regression was deemed appropriate for depression and VL suppression, as they were categorical variables.

A *p*-value < 0.05 was considered statistically significant, with the data being analysed using STATA 16 (StataCorp (2019) Stata Statistical Software: Release 16. StataCorp LLC, College Station, TX).

### Ethical considerations

Ethical clearance to conduct this study was obtained from the University of Kwazulu-Natal, Biomedical Research Ethics Committee (No. BREC/00001085/2020). All potential participants were provided with a description of the study, and written informed consent was obtained from all who volunteered. The participants were allowed to withdraw consent at any stage.

## Results

### Socio-demographic profile

Of the 196 participants, a total of 121 were female (61.7%), 150 were single (76.5%), 131 had no tertiary education (66.8%), and 110 were unemployed (56.1%). The mean age of participants was 39.5 years (standard deviation [s.d.] = 10.6) (Table 1).

**TABLE 1:** Socio-demographic variables of PLWHIV receiving care at HIV clinic.

Variables	*n*	%
**Gender**		
Male	75	38.3
Female	121	61.7
**Age category**		
20–34	66	33.7
35–44	69	35.2
> 45	61	31.1
**Marital status**		
Single	150	76.5
Married	27	13.8
Separated	19	9.7
**Residential area**		
Urban	172	87.8
Rural	24	12.2
**Employment**		
Employed	86	43.9
Unemployed	110	56.1
**Tertiary education**		
Yes	65	33.2
No	131	66.8

PLWHIV, people living with HIV; HIV, human immunodeficiency viruses.

Mean age was 39.5 years (Standard deviation = 10.6).

### Clinical profile

All participants were receiving HAART, the mean CD4 count for the study population being 490.17 (s.d. = 270.86), with 58 participants (33.5%) having a detectable VL (Table 3).

### Adverse childhood experiences

The most common types of ACEs experienced were having one or no parent, parental separation or divorce (*n* = 131, 66.8%), and being exposed to collective violence (*n* = 57, 29.1%) and community violence (*n* = 55, 28.1%). In all, 173 participants (88.3%) experienced ≥ 1 ACE and 79 (40.3%) experienced ≥ 3 ACEs (Table 2).

**TABLE 2:** Clinical characteristics of PLWHIV receiving care at HIV clinic.

Variables	Yes or No	*n*	%	95% CI
Physical abuse	Yes	18	9.2	5.5 14.1
Emotional abuse	Yes	20	10.2	6.3 15.3
Contact sexual abuse	Yes	38	19.4	14.1 25.6
Alcohol and/or drug abuser in the household	Yes	36	18.5	75.4 86.7
Incarcerated household member	Yes	27	13.8	80.6 90.7
Household member treated violently	Yes	51	26.0	84.0 93.2
One or no parents, parental separation or divorce	Yes	131	66.8	59.8 73.4
Emotional neglect	Yes	50	25.5	19.6 32.2
Physical neglect	Yes	20	10.2	6.3 15.3
Bullying	Yes	15	7.7	4.3 12.3
Community violence	Yes	55	28.1	21.9 34.9
Collective violence	Yes	57	29.1	22.8 36.0
ACE ≥ 3	Yes	79	40.3	33.4 47.5

PLWHIV, people living with HIV; HIV, human immunodeficiency viruses; Cl, confidence interval; ACE, Adverse childhood experience.

### Substance use

Thirty-four (17.3%) participant scores on the WHO ASSIST suggested they needed at least a brief intervention for alcohol use, 33 (16.8%) for tobacco use and 13 (6.6%) for cannabis use (Table 3). The lifetime prevalence of cocaine and stimulants was 1.02 (*n* = 2), and no participants used inhalants, sedatives, hallucinogens, opioids, sugars, whoonga or mandrax. The group sizes were, therefore, not large enough for meaningful associations.

**TABLE 3:** Clinical characteristics of PLWHIV receiving care at HIV clinic.

Variables	*n*	%	95% CI
**Depression**			
PHQ-	158	80.6	74.4 85.6
PHQ+	38	19.4	14.4 25.6
**Tobacco risk (ASSIST)**			
No intervention	163	83.2	77.2 87.8
Brief intervention	31	15.8	11.3 21.7
Intensive intervention needed	2	1.0	0.3 4.0
**Alcohol use (ASSIST)**			
No intervention	162	82.7	76.7 87.4
Brief intervention	31	15.8	11.3 21.7
Intensive intervention needed	3	1.5	0.5 4.7
**Cannabis use (ASSIST)**			
No intervention	183	93.4	88.9 96.1
Brief intervention	12	6.1	3.5 10.5
Intensive intervention needed	1	0.5	0.1 3.6
**Viral load**			
Suppressed	115	66.5	59.1 73.2
Unsuppressed	58	33.5	26.9 40.9
**Latest CD4 count**			
Mean (s.d.)	490.17	(270.86)	452.0 528.3

PLWHIV, people living with HIV; HIV, human immunodeficiency viruses; Cl, confidence interval; PHQ-, Screened negative for depressive symptoms; PHQ+, Screened positive for depressive symptoms; CD4, clusters of differentiation 4; s.d., standard deviation.

### Depressive symptoms

Thirty-eight (19.4%) participants screened positive for depressive symptoms (Table 3). A bivariate analysis was conducted to test for association between depression and substance use. There was no association between the depressive risk score and alcohol use risk score (*p* = 0.152), tobacco use risk score (*p* = 0.128), and cannabis use risk score (*p* = 0.428).

### Regression results

Table 4 describes the association between three or more adverse childhood experiences with clinical variables compared to those with less than three adverse childhood experiences. We tested for the association between depression, substance use and VL suppression as outcomes, and the exposure to ≥ 3 ACEs controlling for socio-demographic variables and latest CD4 count. Exposure to ≥ 3 ACEs was associated with greater alcohol (adj β = 2.84, *p* < 0.01), tobacco (adj β = 3.64, *p* < 0.01), and cannabis use risk scores (adj β = 2.39, *p* < 0.01). Similarly, a higher depressive risk (adjusted odds ratio [OR] = 9.39, 95% confidence interval [CI] 4.78–23.51) was also associated with ≥ 3 ACEs. Other notable results included a lower alcohol (adj β = -5.17, *p*≈< 0.01), tobacco (adj β = -4.87, *p* < 0.01) and cannabis use risk scores (adj β = -2.13, *p* < 0.01) among females compared to males (Table 5).

**TABLE 4:** Clinical characteristics of PLWHIV receiving care at HIV clinic by exposure to ACEs.

Variables	ACE < 3		ACE ≥ 3		*df*	χ^2^	*p*
	*n*	%	*n*	%			
**Depression**	-	-	-	-	1	29.3	< 0.001
PHQ-	109	93.20	49	62.00	-	-	-
PHQ+	8	6.80	30	38.00	-	-	-
**Tobacco risk (ASSIST)**	-	-	-	-	2	15.1	0.001
No intervention	107	91.50	56	70.90	-	-	-
Brief intervention	10	8.50	21	26.60	-	-	-
Intensive intervention needed	0	0.00	2	2.50	-	-	-
**Alcohol use (ASSIST)**	-	-	-	-	2	14.2	0.001
No intervention	106	90.60	56	70.90	-	-	-
Brief intervention	11	9.40	20	25.30	-	-	-
Intensive intervention needed	0	0.00	3	3.80	-	-	-
**Cannabis use (ASSIST)**	-	-	-	-	2	11.5	0.003
No intervention	115	98.30	68	86.10	-	-	-
Brief intervention	2	1.70	10	12.70	-	-	-
Intensive intervention needed	0	0.00	1	1.30	-	-	-
**Viral load**	-	-	-	-	1	1.1	0.29
Suppressed	73	69.50	42	61.80	-	-	-
Unsuppressed	32	30.50	26	38.20	-	-	-
**Latest CD4 count†**	-	-	-	-	194	-	0.36
Mean (s.d.)	504.675	280.217	468.684	256.626	-	-	-

PLWHIV, people living with HIV; HIV, human immunodeficiency viruses; ACE, Adverse childhood experience; PHQ-, Screened negative for depressive symptoms; PHQ+, Screened positive for depressive symptoms; CD4, clusters of differentiation 4; s.d., standard deviation.

† , *t* = 0.91.

**TABLE 5:** Association of ACE with depressive, substance use and viral load scores.

Variables	Depression			Tobacco risk (ASSIST)			Alcohol use (ASSIST)			Cannabis use (ASSIST)			Viral load unsuppressed		
	aOR	s.e.	95% CI	adj *β*	s.e.	*p*	adj *β*	s.e.	*p*	adj *β*	s.e.	*p*	aOR	s.e.	95% CI
**ACE ≥ 3**															
No	-	-	-	-	-	-	-	-	-	-	-	-	-	-	-
Yes	9.39	4.40	4.78 23.51	3.64	1.00	< 0.01	2.84	0.99	< 0.01	2.39	0.66	< 0.01	1.27	0.46	0.63 2.58
**Gender**															
Male	-	-	-	-	-	-	-	-	-	-	-	-	-	-	-
Female	1.15	0.53	0.31 2.82	−4.87	1.04	< 0.01	−5.17	1.03	< 0.01	−2.13	0.69	< 0.01	1.43	0.55	0.68 3.02
**Age category (years)**															
20–34	-	-	-	-	-	-	-	-	-	-	-	-	-	-	-
35–44	1.14	0.57	0.26 3.04	−0.96	1.23	0.43	−2.67	1.22	0.03	0.46	0.81	0.57	1.20	0.54	0.49 2.92
> 45	0.64	0.37	−0.76 2.02	−2.43	1.33	0.07	−5.66	1.31	< 0.01	0.06	0.87	0.95	1.26	0.61	0.49 3.28
**Marital status**															
Single	-	-	-	-	-	-	-	-	-	-	-	-	-	-	-
Married	0.47	0.40	−0.90 2.46	1.01	1.50	0.50	1.16	1.49	0.43	0.37	0.99	0.71	0.37	0.23	0.11 1.26
Separated	2.43	1.67	1.30 9.33	−0.89	1.77	0.61	1.49	1.76	0.40	−0.55	1.17	0.64	0.37	0.24	0.11 1.31
**Residential area**															
Urban	-	-	-	-	-	-	-	-	-	-	-	-	-	-	-
Rural	2.26	1.51	1.21 8.39	0.19	1.50	0.90	1.13	1.48	0.45	−0.32	0.99	0.75	0.38	0.23	0.11 1.25
**Employment**															
Employed	-	-	-	-	-	-	-	-	-	-	-	-	-	-	-
Unemployed	1.76	0.75	1.33 4.04	0.75	1.00	0.45	−0.06	0.99	0.95	0.98	0.66	0.14	1.71	0.62	0.84 3.50
**Tertiary education**															
Yes	-	-	-	-	-	-	-	-	-	-	-	-	-	-	-
No	0.45	0.19	−1.84 1.05	−0.05	1.06	0.96	−0.84	1.05	0.42	−1.04	0.70	0.14	1.16	0.43	0.56 2.42
**Latest CD4 Count**	1.00	0.01	0.54 1.00	−0.01	0.01	0.57	−0.01	0.01	0.56	−0.01	0.01	0.70	0.99	0.01	0.99 0.99

ACE, Adverse childhood experiences; aOR, Adjusted odds ratio; s.e., standard error; Cl, confidence interval; CD4, clusters of differentiation 4.

## Discussion

We investigated the prevalence and association of ACEs, substance use and depressive symptoms in PLWHIV during the COVID-19 pandemic at a HIV service clinic in SA. The majority of PLWHIV experienced ≥ 1 ACE and there was a high prevalence of multiple adverse events experienced. The most used substances were alcohol, tobacco and cannabis, which were all significantly associated with experiencing ≥ 3 ACEs. There was a high prevalence of depressive symptoms, with a significant association between depressive symptoms and ≥ 3 ACEs.

The sample of PLWHIV was predominantly female, which is consistent with other studies on PLWHIV in SA,^17,46^ and could be accounted for by their higher vulnerability to HIV infection.^5^ The prevalence of exposure to ≥ 1 ACE (88.3%) by PLWHIV is similar to an international report of 82.5%,^15^ but higher than the findings in SA of 74.8%,^26^ or 68.9%.^47^ Those study population groups did not specifically contain PLWHIV, a cohort more exposed to ACEs than the general population.^48^ The prevalence of exposure to ≥ 3 ACEs was 40.3%, in keeping with the SASH study (38%),^26^ but is lower than PLWHIV internationally (89%).^29^ The pattern of high co-occurrence between multiple ACEs is also consistent with the results found in the general population.^24,27^ The most common ACE experienced was having one or no parents, parental separation or divorce, which was consistent with both international^49^ and local findings.^43^

Our study findings on substances used were consistent with commonly used drugs of abuse at South African treatment centres of alcohol, followed by tobacco and cannabis.^50^ The finding that 17.3% needed at least a brief intervention for alcohol is above general population rates in SA,^1,2^ but lower than findings of some older studies with PLWHIV.^17,46^ The finding that 16.8% of PLWHIV in this study had substance risk scores suggesting the need for a brief intervention for tobacco smoking is lower than previous prevalence findings of 34.0% in SA.^51^ The finding that 6.6% of PLWHIV in this study had cannabis risk scores which suggested the need for at least a brief intervention is similar to the 5.6% finding of PLWHIV meeting criteria for cannabis use disorder,^52^ and higher than the rates in the general population in SA.^53^ This is unsurprising as PLWHIV are more likely to be current cannabis users compared to their negative counterparts.^52,54^ The finding is, however, lower than in international study cohorts of PLWHIV.^55^ These lower findings related to alcohol and tobacco could be influenced by the effects of COVID-19 containment measures with limited accessibility to substances, less social gatherings, fear of the impact of substances on COVID-19 disease severity as well as financial issues. The associations between ≥3 ACEs and alcohol, tobacco and cannabis use risk scores are supported by the literature,^56^ and highlight the negative impact of cumulative ACEs on later substance use risk.

Our finding of 19.4% of PLWHIV screening positive for depressive symptoms is similar to previous reports of 22%,^57^ and in keeping with double the rates expected of the general population in SA.^1^ It is however lower than PLWHIV exposed to ≥4 ACEs internationally (55.6%) and previous local reports,^17,58^ and international reports on PLWHIV during the COVID-19 pandemic.^59^ Based on the literature, a higher prevalence of depressive symptoms during the COVID-19 pandemic could have been anticipated,^6,37^ but possibly attending a clinical service was protective.

The gender differences in substance use indicated by lower alcohol (adj *β* = -5.17, *p* < 0.01), tobacco (adj *β* = -4.87, *p* < 0.01) and cannabis use risk scores (adj *β* = -2.13, *p* < 0.01) among females compared to males, were consistent with previous studies.^1^ The lack of association between depressive symptoms and substance risk scores on WHO ASSIST suggesting intervention is not in keeping with previous reports that indicate an association between them in women living with HIV.^60^ In addition, the association between ACEs and depressive symptoms and problematic substance use in this study suggests that ACEs may be the crucial common factor in driving later substance use behaviour and depression risk, and that public health programmes should therefore review policies and programmes to address this adequately by improving early childhood interventions for losses, providing psychosocial support for families, and legal and psycho-social strategies to address domestic and community violence.

In terms of a significant association between experiencing to ≥3 ACEs and depressive symptoms, our findings are consistent with some studies,^56^ but not in keeping with others^15^ where the majority of participants were male. This suggests that gender differences in this population also need to be explored further.

### Limitations and strengths

There were limitations to this study. This study was cross-sectional, with the relationships identified possibly being bi-directional. Adverse Childhood Experiences were also self-reported retrospectively, with a recall bias possibly being present. Self-report surveys are also subjective and may be biased if social desirability is considered. Data for this study were collected after the first wave of the COVID-19 pandemic, which may have impacted the degree of substance use and depressive symptoms experienced at that time. It was also not possible to determine a causal relationship between pandemic-related exposure and associated outcomes. A follow-up study post pandemic would be beneficial to re-assess these factors. Finally, the study was limited by being based at one clinical site in an urban area which may mean that the results may not be generalisable to other settings. It is, therefore, recommended that the study should also be conducted in a more rural setting. Gender differences can be further explored with regards to types of ACEs experienced, differences in substances used as well as severity of depressive symptoms experienced. The strengths of the study include exploring four variables: ACEs, depressive symptoms, substance use and HIV outcomes in one study, particularly during the pandemic, as a retrospective review of patient-reported symptoms during the pandemic may be less accurate. The study also utilised objective measures that have been previously used in the South African setting.

## Conclusion

Our study findings support the hypothesised cumulative effect of increased exposure to ACEs and association with alcohol, tobacco and cannabis use risk and depressive symptoms in PLWHIV. The high prevalence of substance use and depressive symptoms observed in this study highlights the importance of prioritising and monitoring the mental health needs of this vulnerable population especially in times of disaster such as the COVID-19 pandemic. The findings also support screening for ACEs, substance use and depressive symptoms in PLWHIV as well as highlight the need for larger longitudinal studies that include interventions to address this triple burden of disease. This will ensure that high risk patients can be identified timeously and managed holistically. Psychotherapeutic interventions especially with a cognitive behavioural component, together with targeted pharmacotherapy may be initiated earlier to optimally treat the patients. Integration of psychiatric and psychological services into emergency responses for future pandemics and other crises should be considered in order to reduce any adverse effects on mental health.
